# B7-H1 Expression Is Required for Human Endometrial Regenerative Cells in the Prevention of Transplant Vasculopathy in Mice

**DOI:** 10.1155/2018/2405698

**Published:** 2018-03-14

**Authors:** Kui Ye, Xu Lan, Grace Wang, Baoren Zhang, Xiaoxi Xu, Xiang Li, Yiming Zhao, Hao Wang

**Affiliations:** ^1^Department of General Surgery, Tianjin Medical University General Hospital, Tianjin, China; ^2^Tianjin General Surgery Institute, Tianjin, China; ^3^Department of Vascular Surgery, Tianjin Fourth Central Hospital, Tianjin, China; ^4^Faculty of Medicine, University of Toronto, Toronto, ON, Canada; ^5^Department of Endocrinology, Tianjin Medical University General Hospital, Tianjin, China

## Abstract

Vasculopathy is one of the primary pathological changes in chronic rejection of vascularized allograft transplantation. Endometrial regenerative cells (ERCs) are mesenchymal-like stromal cells with immunosuppressive effect. B7-H1 is a negative costimulator that mediates active immune suppression. The aim of this study was to investigate the requirement of B7-H1 in the immunoregulation of ERCs in preventing transplant vasculopathy of aorta allografts. The results showed that B7-H1 expression on ERCs was upregulated by IFN-*γ* in a dose-dependent manner and it was required for ERCs to inhibit the proliferation of peripheral blood mononuclear cells (PBMCs) *in vitro*. ERCs could alleviate transplant vasculopathy, as the intimal growth of transplanted aorta was limited, and the preventive effects were correlated with an increase in the percentages of CD11c^+^MHC class II^low^CD86^low^ dendritic cells, CD68^+^CD206^+^ macrophages, and CD4^+^CD25^+^Foxp3^+^ T cells, as well as a decrease in the percentages of CD68^+^ macrophages, CD3^+^CD4^+^ T cells, CD3^+^CD8^+^ T cells, and donor-reactive IgM and IgG antibodies. Moreover, overexpression of B7-H1 by IFN-*γ* can promote the immunosuppressive effect of ERCs. These results suggest that overexpression of B7-H1 stimulated by IFN-*γ* is required for ERCs to prevent the transplant vasculopathy, and this study provides a theoretical basis for the future clinical use of human ERCs.

## 1. Introduction

With the rapid development of surgical procedures, organ and cell isolation, and preservation techniques, as well as the advance of transplant immunology, organ transplantation has become an effective and routine treatment for end-stage diseases. Moreover, due to the progression of allograft immunology, some new types of immunosuppressive agents and therapy are used and that have prevented or reversed acute allograft rejection effectively in the past 20 years, which lead to the short-term (1-year) allograft survival time of 88–95% [1]. However, these immunosuppressive therapies are not effective for the prevention and treatment of chronic rejection caused by transplant vasculopathy. Therefore, chronic rejection with the vascularized allografts causing late allograft failure remains a clinical challenge [2]. Vasculopathy is the basic pathological change in chronic rejection and caused by complex interplay between immune and nonimmune factors, which can lead to tissue ischemia of transplanted organs and subsequently organ failure [3]. Thus, the transplant vasculopathy in chronic rejection has become a major obstacle to long-term survival of transplanted organs, and an effective treatment is urgently needed [4].

Endometrial regenerative cells (ERCs) obtained from human menstrual blood are present as a kind of attractive mesenchymal-like stromal cells and have been demonstrated to show high immunosuppressive activities in mouse models of organ transplantation and other inflammation [5–8]. Compared to mesenchymal stromal cells (MSCs), ERCs have the advantages of noninvasively obtained method, body waste reused, abundant resources, highly proliferative rate, pluripotent differentiation activity, expandability to great quantities without karyotypic abnormalities, or the loss of differentiation ability, and without oncogenesis [9]. Recently, our group had demonstrated that ERCs could suppress B cell proliferation and further suppress the humoral response to allogenic cardiac transplants in mice [8]. As transplant vasculopathy is a kind of allograft rejection, there is a need to investigate the effect of ERCs on the treatment of it.

B7-H1, also known as programmed cell death-1 ligand (PD-L1), as a member of the B7 family, has been well identified to link to the active immunosuppression [10]. It has been demonstrated that B7-H1 on MSCs can be upregulated in response to the stimulation of proinflammatory interferon- (IFN-) *γ* and it is required in MSC-based therapy for inducing immune tolerance to cardiac allografts by inducing the generation of regulatory immune cells such as tolerogenic dendritic cells (Tol-DCs), regulatory T cells (Tregs), and regulatory B cells (Bregs), and inhibiting the generation of B cells and allogenic antibodies [11], suggesting that B7-H1 plays a critical role in the maintenance of peripheral tolerance.

However, the role of B7-H1 on ERC-mediated immunoregulation has not been investigated. Thus, the objective of this study was to determine whether the expression of B7-H1 on ERCs can be upregulated by the stimulation of IFN-*γ* and to determine the role of ERC-expressing B7-H1 in preventing transplant vasculopathy in aorta allografts by using neutralizing anti-B7-H1 monoclonal antibody (mAb).

## 2. Materials and Methods

### 2.1. Animals

Male adult C57BL/6 (B6) (H-2^b^) and BALB/c (H-2^d^) mice (10–12 weeks old and 18–22 g in weight) were purchased from China Food and Drug Inspection Institute (Beijing, China). The animals were housed under a conventional experimental environment at Tianjin General Surgery Institute (Tianjin, China) and provided with water and chow ad libitum. All the experiments were performed on the basis of protocols approved by the Animal Care and Use Committee of Tianjin Medical University (Tianjin, China), according to the Chinese Council on Animal Care guidelines, with IRB number IRM-201405-071.

### 2.2. Isolation, Culture, and Pretreatment of ERCs

The menstrual blood samples from one 30-year-old healthy woman were collected by using a sterilized menstrual cup on the first day of menstruation for isolation of ERCs under ethical approval of Tianjin Medical University (Tianjin, China), with IRB number IRB2017-YX-094. The ERC culture and expansion were the same as described previously [8]. In brief, after being collected, the mononuclear cells in the menstrual blood samples were isolated using standard Ficoll (Solarbio, Beijing, http://www.solarbio.net.cn), then transferred to a culture dish with Dulbecco's modified Eagle's medium (DMEM) high glucose containing 1% penicillin/streptomycin and 10% FBS, and cultured in a 37°C 5% CO_2_ incubator. The morphology and growth of the cultured cells were observed under inverted microscope. After about 14 days of incubation, the cells displayed a spindle-shaped morphology and the estimated adherent cell number at the start of culture was approximately 1 × 10^7^. Typical cell surface markers of ERCs were analyzed by using a fluorescence-activated cell sorting (FACS) analysis as previously described [9]. In order to investigate the effect of IFN-*γ* on the expression of B7-H1 on the surface of ERCs *in vitro*. The ERCs of passage four were harvested, counted, and adjusted to 1 × 10^5^/ml cell concentration, then added to a 4-well plate and incubated with 1 ml of the culture medium containing recombinant human IFN-*γ* (PeproTech, Jiangsu, China; https://www.peprotech.com) at different concentrations (0.05 ng/ml, 0.5 ng/ml or 5 ng/ml). After 72 hours of incubation, ERCs were collected and labeled with anti-human B7-H1 antibody for the FACS analysis. For the *in vivo* use, the fourth generation of ERCs was either cultured in the culture medium with IFN-*γ* (5 ng/ml) and anti-B7-H1mAb (Clone M1H2, Thermo Fisher Scientific, Shanghai, China; https://www.thermofisher.com/cn/zh/home.html) or untreated for 72 hours. After that, the ERCs were collected and washed for further *in vivo* using.

### 2.3. Cocultures of ERCs with Allogeneic Peripheral Blood Mononuclear Cells (PBMCs)

In order to examine the function of B7-H1 expressed on ERCs, the coculture experiments were performed between the fourth generation of ERCs pretreated with either IFN-*γ* and anti-B7-H1 mAb or nothing and B6 PBMCs. ERCs (5 × 10^4^ cells) as stimulators were then pretreated with mitomycin C (50 *μ*g/ml) (Solarbio, Beijing, China, http://www.solarbio.net.cn), and PBMCs (5 × 10^5^ cells) were responders. After 72-hour incubation, the proliferative response of PBMCs to the ERCs was examined assessed by Cell Counting Kit-8 (CCK-8) (Dojindo, Shanghai, China, http://www.dojindo.cn) through the Microplate Reader. The optical density (OD) value at 450 nm was recorded.

### 2.4. Aorta Transplantation and Experimental Groups

A 3-4 mm segment of the descending abdominal aorta was collected from male BALB/c(H-2^d^) donors and was transplanted to the same location of fully MHC mismatched male C57BL/6 (B6) (H-2^b^) recipients with the embedded sleeve method using 11–0 monofilament nylon sutures [12]. The success of the graft was determined by the presence of both the hind legs working well without paralysis, which was monitored and evaluated daily in double-blind fashion. After the surgery, the recipients were randomly assigned to four groups (*n* = 6, each group): group 1, untreated group (untreated); group 2, anti-B7-H1 antibody-pretreated ERC group; group 3, ERC-treated group; group 4, IFN-*γ*-pretreated ERC group. The ERCs pretreated with different reagents or not were intravenously injected into B6 recipients through the tail vein with a single dose of 1 × 10^6^ cells/mouse 24 hours after aorta transplantation.

### 2.5. Graft Histology

Transplanted aorta allografts were collected at the posttransplantation day 40, followed by formalin (10%) fixation and paraffin embedding. Sections (4 *μ*m) of graft tissues were stained with hematoxylin and eosin (H&E) and examined in a blinded fashion for severity of rejection by a pathologist. The intimal thickness of the grafts was measured by the ImageJ software (National Institutes of Health, Bethesda, MD, USA).

### 2.6. FACS Analysis

The spleens from mice in each group were collected at posttransplantation day 8, after being grinded and filtered; and after lysing of the red blood, the splenocytes were washed and dispensed for using. FACS analysis was performed to determine splenocyte phenotypes and donor-reactive antibodies as previously described [11]. All the fluorescent-labeled antibodies against CD3, CD4, CD8, CD11c, CD25, CD68, CD86, CD206, Foxp3, IgM, IgG, and MHC class II were purchased from either eBioscience (eBioscience, San Diego, CA, http://www.eBioscience.com) or BioLegend (BioLegend, San Diego, CA, http://www.biolegend.com).

### 2.7. Mixed Lymphocyte Reaction (MLR)

In order to assess the function of DCs in each group, T cell proliferation response to alloantigen was assessed in a one-way MLR in 96-well plates. Alloreactive CD3^+^ T cells (5 × 10^5^ cells/well) from the spleen of BALB/c mice were used as responders, and splenic CD11c^+^ DCs (5 × 10^4^ cells/well) from B6 recipients were used as stimulators. Both DCs and T cells were isolated from the spleens using a positive microbead-based selection (Miltenyi Biotec Inc., Shanghai, China; http://www.miltenyibiotec.com.cn/) by either CD11c or CD3 MicroBeads. After being selected, the DCs were treated with mitomycin C (50 *μ*g/ml) to inhibit their proliferative function. The proliferative response of T cells to the stimulator of DCs was examined after 96 hours of incubation and compared to those without DCs and then was assessed by CCK-8 through the microplate reader. The OD value at 450 nm was recorded.

### 2.8. Statistical Analysis

The enumeration data were presented as mean ± standard deviation (SD). The differences among multiple groups were analyzed using one-way analysis of variance (ANOVA) followed by post hoc analysis with the least significant difference (LSD) test. A *p* value < 0.05 was considered statistically significant.

## 3. Results

### 3.1. B7-H1 Expression on the ERCs Is Upregulated by IFN-*γ* In Vitro

**Figure 1:**
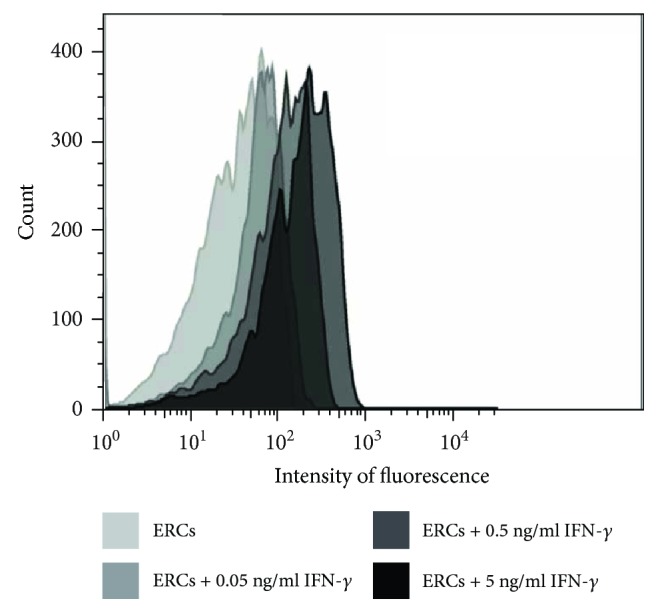
B7-H1 expression on the ERCs is upregulated by IFN-*γ in vitro*. The fourth generation of ERCs was digested, counted, and adjusted to 1 × 10^5^/ml cell concentration and then stimulated in the presence or absence of 0.05 ng/ml, 0.5 ng/ml, or 5 ng/ml of recombinant human IFN-*γ* for 72 hours. B7-H1 expression was analyzed by FACS analysis. The intensity of fluorescence-labeled B7-H1 on the surface of ERCs was shown.

In order to confirm the effect of IFN-*γ* on the expression of B7-H1 on ERCs, cultured ERCs in response to different concentrations of recombination human IFN-*γ* were examined. As shown in Figure 1, a basal level of B7-H1 expression on ERCs (35.9%) was recorded. In addition, with the increased level of IFN-*γ* concentration, the level of B7-H1 expression was upregulated in a dose-dependent manner (64.6% with 0.05 ng/ml IFN-*γ* and 81.4% with 0.5 ng/ml IFN-*γ*) and was at the highest level when stimulated with 5 ng/ml IFN-*γ* (90.4%). As the result showed that much more B7-H1 expression on the ERCs was presented by the treatment with IFN-*γ* at the dose of 5 ng/ml, the ERCs pretreated with 5 ng/ml IFN-*γ* were used as an “upregulated B7-H1” group in the follow-up *in vivo* experiment.

### 3.2. B7-H1 Is Required for ERCs to Inhibit the Proliferation of PBMCs In Vitro

**Figure 2:**
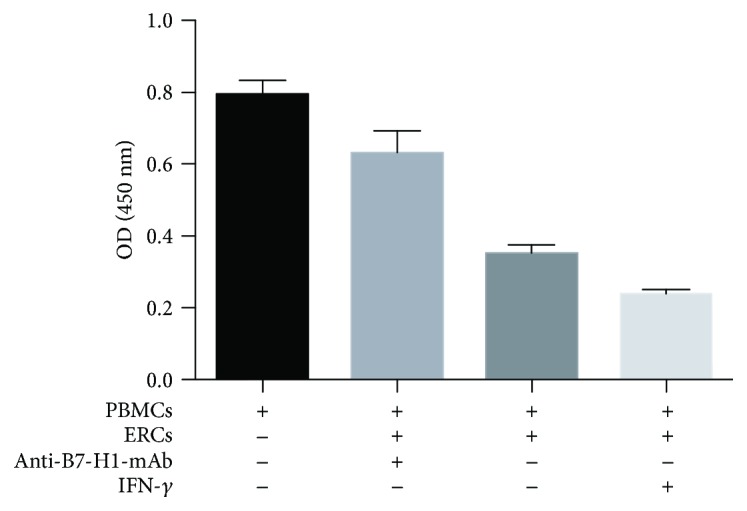
B7-H1 is required for ERCs to inhibit the proliferation of PBMCs *in vitro*. The ERCs pretreated with either IFN-*γ* and anti-B7-H1 mAb or nothing were pretreated with mitomycin C and cocultured with B6 PBMCs for 72 hours. The proliferation of PBMCs was measured by CCK-8, and the OD values of each group were showed. Values were presented as mean ± SEM; statistical analysis was done by one-way ANOVA followed by the LSD test, *n* = 6.

In order to further explore the function of B7-H1 expressed on the ERCs, the coculture experiment was performed and measured by the proliferation of PBMCs through OD value. As shown in Figure 2, same as the previous study [13], compared to the PBMCs alone, ERCs could significantly inhibit the proliferation of PBMCs (*p* < 0.001) and the IFN-*γ*-pretreated ERCs could further inhibit the proliferation of PBMCs (*p* < 0.001). However, when the function of B7-H1 was inhibited by anti-B7-H1 mAb, the proliferation of PBMCs significantly increased compared to that of the ERC groups without inhibition by anti-B7-H1 mAb (*p* < 0.001). The results demonstrated that B7-H1 participated in the ERC-mediated suppression of the PBMCs' proliferation.

### 3.3. B7-H1 Is Required for ERCs to Alleviate Transplant Vasculopathy

**Figure 3:**
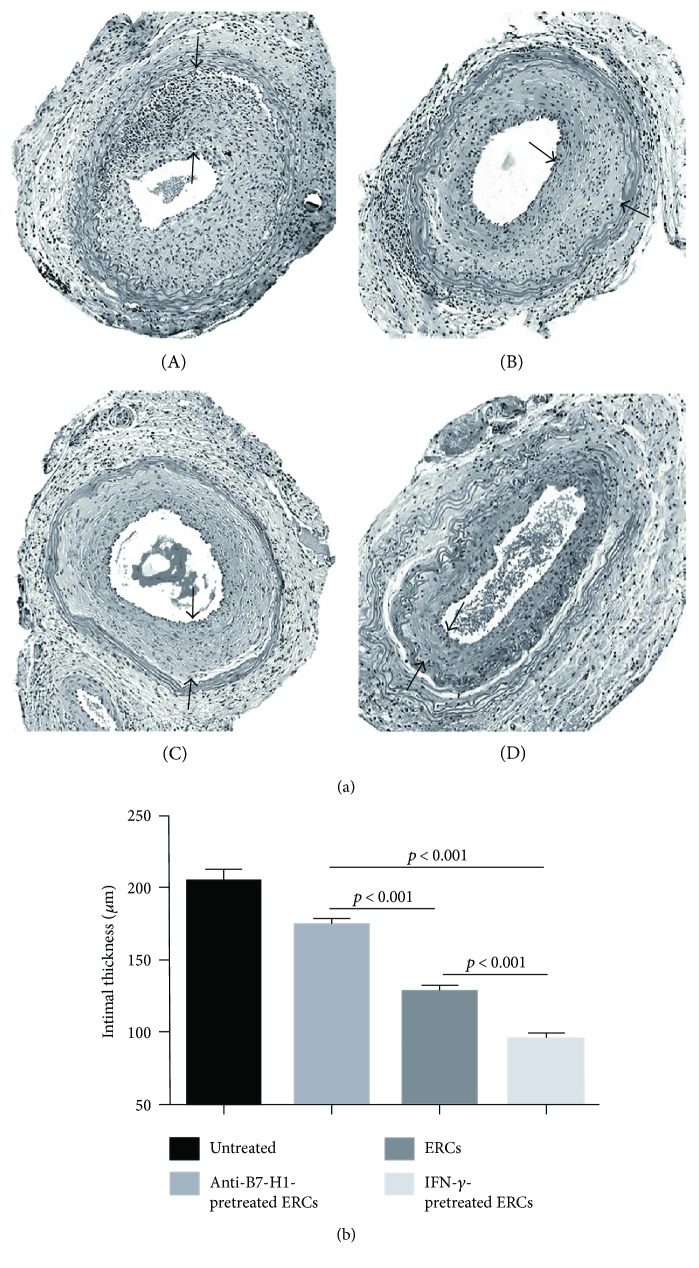
B7-H1 is required for ERCs to alleviate transplant vasculopathy. (a) Histology of the transplanted aorta in B6 recipients. Grafts were collected at posttransplant day 40 and evaluated by H&E staining of paraffin sections. Pathological changes from the (A) untreated group, (B) anti-B7-H1 mAb-pretreated ERC group, (C) ERC-treated group, and (D) IFN-*γ*-pretreated group were presented (*n* = 6). The black arrows indicated the intimal limits (100x magnification). (b) The intimal thickness of the grafts in each group. Values were presented as mean ± SEM; statistical analysis was done by one-way ANOVA followed by the LSD test, *n* = 6.

The typical character of transplant vasculopathy is shown by the diffused concentric intimal expansion of the arteries in grafts. The effects of B7-H1 expression on ERCs in the development of transplant vasculopathy were assessed by observing the intimal growth of the transplanted aorta by H&E staining at posttransplantation day 40. As shown in Figure 3, the thickest intimal (204.11 ± 8.53 *μ*m) was developed in the untreated group with significant immune cell infiltration, which was similar to that of the anti-B7-H1 mAb-pretreated ERC group (174.15 ± 4.47 *μ*m). The ERC treatment markedly decreased the intimal thickness (127.09 ± 5.25 *μ*m, *p* < 0.001 versus the untreated and anti-B7-H1 mAb-pretreated ERC groups), and the IFN-*γ*-pretreated ERC group had the thinnest intimal thickness (94.65 ± 4.88 *μ*m, *p* < 0.001 versus the untreated, anti-B7-H1 mAb-pretreated ERC, and ERC alone groups). The results may suggest that ERCs can reduce the severity of transplant vasculopathy, and the higher B7-H1 expression, the stronger reduction effect of ERCs was observed.

### 3.4. B7-H1 Is Required for Human ERCs to Increase the Population of Tol-DCs in Mice

**Figure 4:**
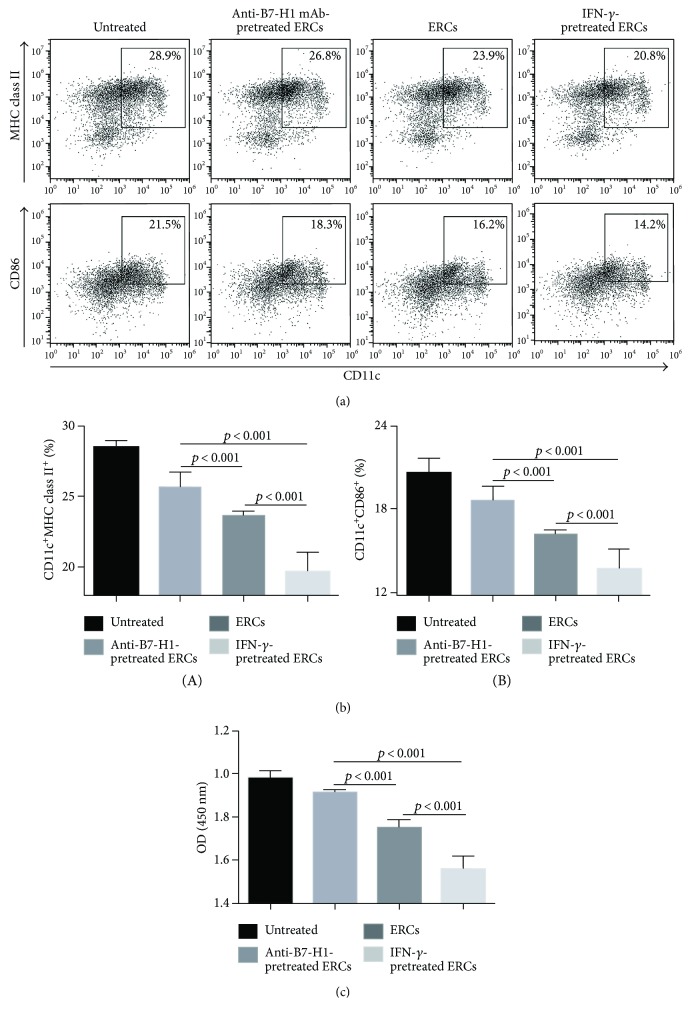
B7-H1 is required for ERCs to increase the percentage of Tol-DCs in splenocytes. Splenocytes were collected from B6 recipients at posttransplant day 8, followed by double staining of anti-mouse CD11c antibody in combination with anti-mouse MHC class II or CD86 antibody for the FACS analysis. (a) Dot plots of (A) CD11c^+^MHC class II^+^DC and (B) CD11c^+^CD86^+^DC in each group. (b) Percentages of CD11c^+^MHC class II^+^DC and CD11c^+^CD86^+^DC in each group. (c) One-way MLR was used to measure the function of CD11c^+^ DCs (stimulator) from B6 recipients in each group. The proliferation index of CD3^+^ T cell (responders) was performed by the OD value. Values were presented as mean ± SEM; statistical analysis was done by one-way ANOVA followed by the LSD test, *n* = 6.

We have recently demonstrated that ERCs could suppress the maturation of DCs and induce the generation of Tol-DCs [6]. In order to understand the role of B7-H1 on the effect of ERCs, we measured the population of Tol-DCs with double staining using anti-mouse CD11c antibody in combination with anti-mouse MHC class II or CD86 antibody in the splenocytes of each group through FACS analysis. As shown in Figure 4, compared to the untreated group and the anti-B7-H1 mAb-pretreated ERC group, the ERC alone group showed significant low expressions of antigen presenting molecule MHC class II and costimulatory molecule CD86 on CD11c^+^ DCs (*p* < 0.001). The CD11c^+^MHC class II^+^ and CD11c^+^CD86^+^ populations were further decreased in the IFN-*γ*-pretreated ERC group (*p* < 0.001 versus the untreated, anti-B7-H1 mAb-pretreated ERC, and ERC alone groups), indicating that human ERCs increase the population of Tol-DCs in mice and B7-H1 expression is required for this process.

At the same time, one-way MLR was performed through measuring the index of antigen-stimulated CD3^+^ T cell proliferation from splenocytes of BALB/c mouse, in order to confirm the function of CD11c^+^ DC populations from B6 recipients of each group. As shown in Figure 4(c), T cell proliferation was significantly reduced in the ERC-treated group (*p* < 0.001 versus the untreated and anti-B7-H1 mAb-pretreated ERC groups) and was further inhibited in the IFN-*γ*-pretreated ERC group (*p* < 0.001 versus the untreated, anti-B7-H1 mAb-pretreated ERC, and ERC alone groups). However, the inhibitory effect of ERCs was impaired when ERCs were pretreated with anti-B7-H1 mAb and the T cell proliferation was indistinguishable from that of the untreated group. Taken together, the data showed that B7-H1 is required for ERCs in enhancing the population and function of Tol-DCs.

### 3.5. B7-H1 Is Required for Human ERCs to Decrease the Population of Total Macrophages and Increase the Percentage of M2 in Mice

**Figure 5:**
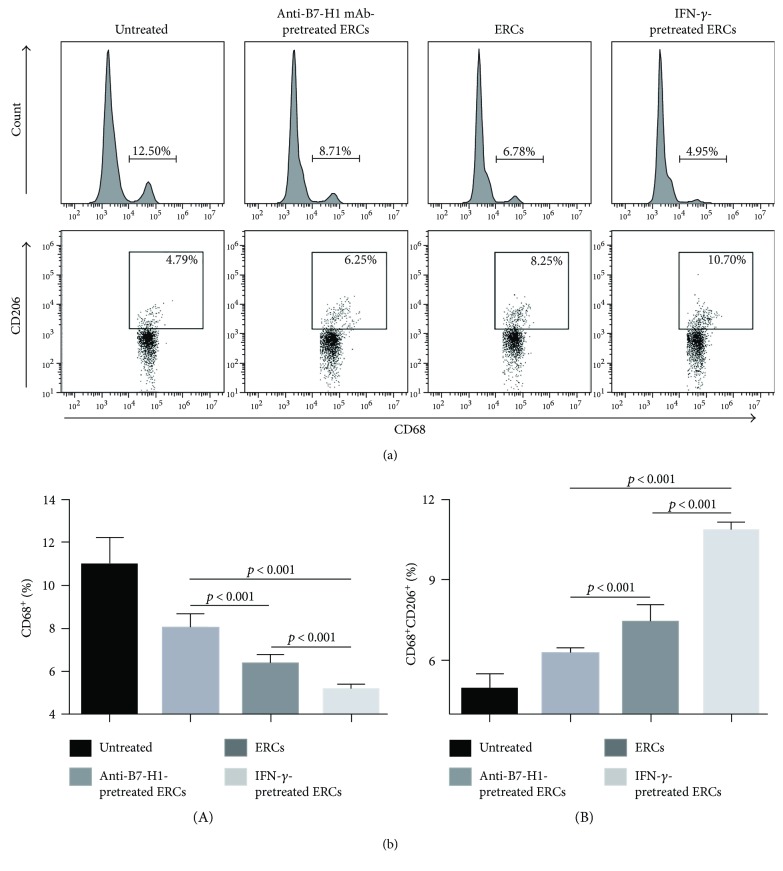
B7-H1 is required for ERCs to regulate the percentages of total macrophages and M2. Splenocytes were collected from B6 recipients at posttransplant day 8, followed by single staining with anti-mouse CD68 antibody to measure the percentage of total macrophages, and together with anti-mouse CD206 antibody gating by CD68 to measure the percentage of M2, the FACS analysis was used. (a) Histograms of CD68^+^ total macrophages and dot plots of CD68^+^CD206^+^ M2. (b) Percentages of CD68^+^ total macrophages and CD68^+^CD206^+^ M2 in each group. Values were presented as mean ± SEM; statistical analysis was done by one-way ANOVA followed by the LSD test, *n* = 6.

The populations of total macrophages and M2 in the recipient splenocytes were compared between groups by FACS analysis. The total macrophages were single stained with anti-mouse CD68 antibody, and the M2 were considered as CD206 positive cells in total macrophages. As shown in Figure 5, ERCs, either unmodified or stimulated by IFN-*γ*, expressed high level of B7-H1 and significantly reduced the population of total macrophages and increased the percentage of M2 as compared to the untreated group (total macrophages: *p* < 0.001; M2: *p* < 0.001). However, the effect of ERCs was abolished when B7-H1 expression was blocked by anti-B7-H1 mAb, as compared to the unmodified ERC-treated group and IFN-*γ*-pretreated ERC group (total macrophages: *p* < 0.001; M2: *p* < 0.001). These results mean that B7-H1 is required for ERCs to induce the macrophage development towards immune tolerance.

### 3.6. B7-H1 Is Required for Human ERCs to Decrease the Population of T Cells and Increase the Percentage of Tregs in Mice

**Figure 6:**
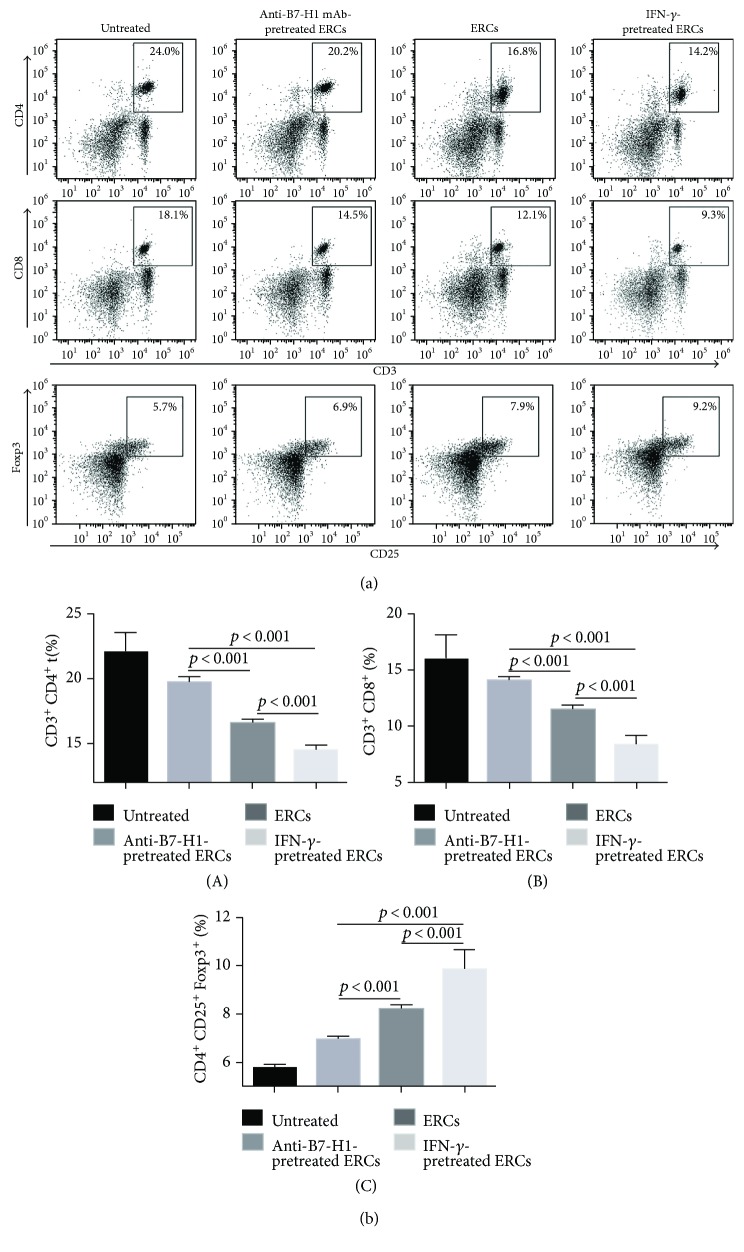
B7-H1 is required for ERCs to regulate the percentages of T cells and Tregs. Splenocytes were collected from B6 recipients at posttransplant day 8, after being stained by fluorescence-labeled antibodies (for T cells were anti-mouse CD3, CD4, and CD8 antibodies; for Tregs were anti-mouse CD4, CD25, and Foxp3 antibodies); the FACS analysis was used. (a) Dot plots of CD3^+^CD4^+^ T cells, CD3^+^CD8^+^ T cells, and CD4^+^CD25^+^Foxp3^+^ T cells in each group. (b) Percentage of CD3^+^CD4^+^ T cells, CD3^+^CD8^+^ T cells, and CD4^+^CD25^+^Foxp3^+^ T cells in each group. Values were presented as mean ± SEM; statistical analysis was done by one-way ANOVA followed by the LSD test, *n* = 6.

In our previous studies, we have demonstrated that ERCs could inhibit the generation of both CD4^+^ T cells and CD8^+^ T cells, as well as promote the generation of Tregs [6, 7]. In this study, the same results were presented and the role of B7-H1 on ERCs in T cell development was explored. The CD3^+^ T cells were isolated from the splenocytes of recipients by the anti-mouse CD3 antibody, and combination with either anti-mouse CD4 antibody or anti-mouse CD8 antibody was used to measure CD4^+^ or CD8^+^ T cells. The Tregs were double staining with anti-mouse CD25 and Foxp3 antibodies after being gated by anti-mouse CD4 antibody. The results were shown in Figure 6; compared to the unmodified ERC-treated group, the IFN-*γ*-pretreated ERC group showed less populations of both CD4^+^ and CD8^+^ T cells and more percentage of Tregs (CD4^+^ T cells: *p* < 0.001; CD8^+^ T cells: *p* < 0.001; Tregs: *p* < 0.001). When B7-H1 on ERCs was blocked by anti-B7-H1 mAb, the opposite results showing increased population of CD4^+^ and CD8^+^ T cells, but a decreased level of Tregs, were found in mouse recipients (CD4^+^ T cells: *p* < 0.001; CD8^+^ T cells: *p* < 0.01; Tregs: *p* < 0.001). These data suggested that B7-H1 is required for ERCs in inhibition of T cells and promotion of Treg expansion.

### 3.7. B7-H1 Is Required for ERCs to Decrease the Serum Levels of Donor-Reactive IgM and IgG in Mice

**Figure 7:**
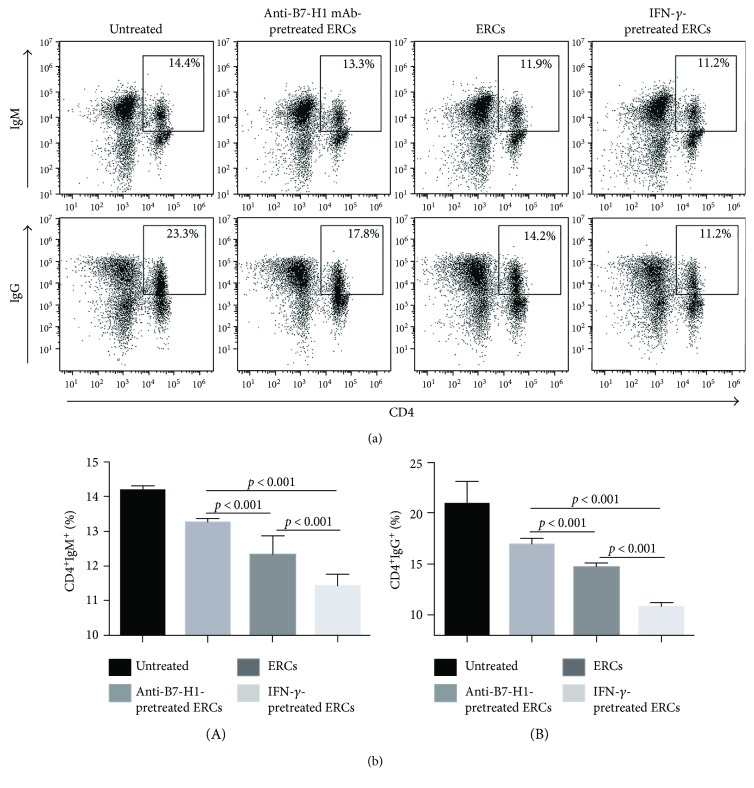
B7-H1 is required for ERCs to decrease the percentage of donor-reactive antibodies in sera. The sera were collected from B6 recipients at posttransplant day 8, 1 : 20 dilution in PBS, then incubated with splenocytes of BALB/c mouse for 30 min at 37°C, and followed by double staining with either anti-mouse CD4 and IgM antibodies or anti-mouse CD4 and IgG antibodies for the FACS analysis. (a) Dot plots of CD4^+^IgM^+^ antibodies and CD4^+^IgG^+^ antibodies in each group. (b) Percentage of CD4^+^IgM^+^ antibodies and CD4^+^IgG^+^ in each group. Values were presented as mean ± SEM; statistical analysis was done by one-way ANOVA followed by the LSD test, *n* = 6.

Circulating donor-reactive IgM and IgG are antibodies produced by B cells and associated with antibody-mediated rejection (AMR) in transplant rejection. These antibody levels in the sera were measured by double staining with either anti-mouse CD4 and IgM antibodies or anti-mouse CD4 and IgG antibodies and analyzed by FACS as described previously [11]. As shown in Figure 7, percentages of circulating anti-donor IgM and IgG antibodies were reduced in the ERC-treated group compared with anti-B7-H1 mAb-pretreated group (IgM: *p* < 0.001; IgG: *p* < 0.001). In the IFN-*γ*-pretreated ERC group, percentages of circulating anti-donor IgM and IgG antibodies were further reduced compared with the unmodified ERC-treated group (IgM: *p* < 0.001; IgG: *p* = 0.016). The results indicated that ERCs could decrease the generation of circulation antidonor antibodies and B7-H1 expressed on ERCs was critical in this process.

## 4. Discussion

Chronic rejection is a major factor affecting long-term survival of the transplanted organs, and the transplant vasculopathy is the typical manifestation. Although the pathogenesis of vasculopathy is complicated, endothelial injury and dysfunction and inflammatory cell infiltration are common pathological features [3]. Both cellular immunity and humoral immunity are involved in this process.

ERCs were first identified in 2007 [14]. They are mesenchymal-like stromal cells with the immunoregulatory function and have been demonstrated to play protective roles in murine inflammatory and transplantation models [5–8, 15, 16]. However, the mechanism of its function needs to be further clarified. B7-H1 was first identified in 1999 and supposed to be a negative costimulator, which could stimulate T cells to release interleukin- (IL-) 0, suppress T cell proliferation, and activate immune suppression in the immune response [10], with its receptor being programmed cell death receptor 1 (PD-1). The B7-H1/PD-1 pathway commonly plays an immune suppressive effect which has been well demonstrated in tumor and inflammatory immunity [17–19]. Some cytokines can induce the higher expression of B7-H1, such as IFN-*γ*, IL-10, and vascular endothelial growth factor (VEGF), of which IFN-*γ* is the most potent one [20–23]. In the related study of MSCs, IFN-*γ* can act directly and upregulate B7-H1 on MSCs and further trigger the immunosuppressive effect of MSCs [24, 25]. ERCs and MSCs have many similarities in their character; thus, we carried out this study to investigate the role of B7-H1 on the surface of ERCs in an aorta transplant model. On the basis of previous studies of ERCs, through upregulating B7-H1 by IFN-*γ* and neutralization of B7-H1 by anti-B7-H1 mAb, the present study has demonstrated that B7-H1 plays a critical role in enhancing the immunosuppressive effects of ERCs both *in vitro* coculture cell proliferation and *in vivo* transplant vasculopathy. We have also demonstrated that ERCs attenuated the pathological changes in transplant vasculopathy, which was further alleviated by the IFN-*γ*-pretreated ERCs. The immunosuppressive effect of ERCs with upregulated expression of B7-H1 is related to the increase of tolerogenic cells (Tol-DCs, M2, and Tregs), the decrease of invasive cells (total macrophages, CD4^+^ T cells, and CD8^+^ T cells), and the downregulation of serum donor-reactive antibodies (IgM and IgG antibodies).

DCs are the main antigen-presenting cells (APCs), which come into effect through high-expressing antigen-presenting molecule MHC class II and costimulatory molecule CD86 on their surface [26]. On the contrary, Tol-DCs are defined as low expression of these molecules which represents low antigen presenting capability and strong immunosuppressive capability [26]. Macrophages are the most plastic cells in the innate immune system; its differentiation is affected by the surrounding environment [27]. M2 is a small group of the macrophages; they often contribute to the immunoregulatory function [28]. T cells play roles in cellular immunity, and B cells play roles in humoral immunity mainly by secreting antibodies. As the other solid organ transplantation, most of the rejection pathological processes begin with the damage of the endothelial cells caused by immune cells and donor-reactive antibodies. As the foreign antigens were expressed on the endothelial cells of the allografts, the innate and adaptive immune cells of recipients would recognize and attract them directly by secreting cytokines, such as IL-2, IL-6, IFN-*α*, and TNF-*α* [3]. At the same time, donor-reactive antibodies can bind to the antigens on the surface of endothelial cells; then some immune reactions will happen [29]. They can activate the complement system and eventually lead to the formation of the membrane attack complex (MAC) or bind to and activate the innate immune cells like macrophages and lead to the happening of antibody-dependent cell-mediated cytotoxicity (ADCC) or promote exocytosis of the Weibel-Palade bodies, activate the coagulation cascade, and increase the expression of adhesion molecules and growth factors [30]. In a word, both cellular and humoral immunity could lead to the damage of endothelial cells, and then the smooth muscle cells from the media migrate to the intima with the phenotype change; finally, the intimal fibroproliferative growth is initiated, accompanied by immune cell infiltration [31–33].

The key point of alleviating the severity of transplant vasculopathy is to regulate these immune cells and antibodies. Our study presented that ERCs came into an immunosuppressive effect in the transplant vasculopathy, mainly through regulating the percentage of these immune cells and antibodies. At the same time, all of these immune cells express PD-1, the receptor of B7-H1 [21, 34–39]. When the function of B7-H1 on the ERCs was inhibited by the anti-B7-H1 mAb, the effect of ERCs was attenuated. This suggests that B7-H1 plays an essential role in mediating ERC function, and upregulation of B7-H1 on the ERCs may direct immune cell generation and function to the immunoregulatory types. Thus, we upregulated the expression of B7-H1 on the ERCs by being stimulated with IFN-*γ*; the results were the same as what we assumed that B7-H1 expressed on the surface of ERCs could increase the ERC immunoregulatory effect and further alleviated the transplanted vasculopathy.

Although the immunoregulatory mechanisms of ERCs are complex, our results have demonstrated that B7-H1, at least in part, plays an important role in ERC-mediated immunosuppressive effect.

## 5. Conclusions

In conclusion, ERCs can prevent the transplant vasculopathy as the intimal growth of transplanted aorta is inhibited. This effect is achieved via increasing the populations of tolerogenic cells, as well as decreasing the percentage of invasive cells and serum levels of donor-reactive antibodies. Moreover, B7-H1 plays a critical role in the effect of ERCs and heightening the expression of B7-H1 by IFN-*γ* can promote the immunosuppressive effect of ERCs. Previous clinical studies have shown that ERCs are safe to be used without the happening of immunological reactions [16, 40, 41]. This study explores the initial role and mechanism of ERCs in attenuating transplant vasculopathy following aorta allotransplantation, and it may provide a theoretical basis for the future clinical use of ERCs in preventing chronic allograft rejection.
